# Enabling Efficient Communications with Resource Constrained Information Endpoints in Smart Homes

**DOI:** 10.3390/s19081779

**Published:** 2019-04-13

**Authors:** Diego Sánchez-de-Rivera, Borja Bordel, Ramón Alcarria, Tomás Robles

**Affiliations:** Universidad Politécnica de Madrid, UPM Campus Sur, Km 7.5 de la Autovía de Valencia, 28031 Madrid, Spain; diegosanchez@dit.upm.es (D.S.-d.-R.); ramon.alcarria@upm.es (R.A.); tomas.robles@upm.es (T.R.)

**Keywords:** resource consumption, Smart Homes, information endpoints, mathematical models, efficiency, dynamic configuration

## Abstract

Smart Homes are one of the most promising real applications of Internet of Things and Cyber-Physical Systems. Devices and software components are deployed to create enhanced living environments where physical information is captured by sensors, sent to servers and finally transmitted to information endpoints to be consumed after its processing. These systems usually employ resource constrained components in dense architectures supported by massive machine type communications. Components, to adapt to different scenarios, present several configuration options. In machine type communications, these configuration options should be selected dynamically and automatically. Many works have addressed this situation in relation to sensor-server communications but endpoints are still mostly manually configured. Therefore, in this paper it is proposed an automatic and dynamic configuration algorithm, based on the idea of “efficiency,” for information endpoints in the context of Smart Homes. Different costs associated to endpoint-server communications in Smart Homes are identified and mathematically modelled. Using this model and real measurements, the most efficient configuration is selected for each endpoint at each moment, not only guarantying the interoperability of devices but also ensuring an adequate resource usage, for example, modifying the endpoints’ lifecycle or the information compression mechanism. In order to validate the proposed solution, an experimental validation including both real implementation and simulation scenarios is provided.

## 1. Introduction

Smart Homes [1] are domestic technology enhanced living environments supported by pervasive sensing platforms, information servers and information endpoints interacting with inhabitants [2]. The idea of “Smart Homes” is quite old but it has been adapted to different technological paradigms over the years [3]. Nowadays, most modern deployments are based on Internet of Things [4] architectures and Cyber-Physical Systems [5], where physical and computational processes interact and evolve together. In that way, Smart Homes are one of the most promising application scenarios of future engineered systems.

Smart phones, passive and active tags, personal computers and so forth, have been employed to build Smart Homes, although currently most typical deployments follow a common scheme [6]. First, sensors (and probably actuators) capture information about the environment. Sensors include [7] video tracking systems, wrist actigraph units, infrared sensors, ultrasonic devices, microphones and so forth. All this information is collected and sent to a server (or probably a distributed solution) where it is aggregated and processed. This server maintains components describing and controlling the entire system behaviour. Prosumer environments, temporal logic, rules, executable processes and other similar technologies [8] may be employed to enable users to define the expected behaviour and the help policies they desire; as the final objective of every Smart Home is helping people in their daily living activities. Finally, although a lot of information is exclusively collected to make decisions in the server, sometimes information is sent to information endpoints where it is consumed. Displays or speakers are examples of information endpoints [9]. The objective of these elements is to interact with people, making them aware about certain relevant information (collected from sensors or generated in the server).

Both, sensors and endpoints, tend to be resource constrained devices in order to adapt the investment as much as possible to the users’ needs. Hundreds or even thousands, of resource constrained components are integrated into dense technological deployments, without external power supply (devices usually employ batteries) or communication infrastructure but communicating through Massive Machine Type Communications (MMTC) [10], which are solutions to automatically create communication links among devices. To make viable these new solutions, two conditions are usually considered: (i) sensors and endpoints must be interoperable with any server or Smart Home and (ii) devices’ lifetime must be large enough compared to the system lifetime. The first condition is met by including in every device many different configuration options; and the second one requires from managers to configure components to make the most efficient possible use of resources. In this context, a “configuration option” is understood as a particular set of values for the variables that control the behaviour of the device (endpoint). For example, the transmission protocol or the update period to refresh the displayed information. 

As MMTC are automatic technologies, the (dynamic) configuration process in sensors and endpoints must be automatic and fulfil the efficiency condition. Many different authors and works have addressed this problem in relation to sensor-server communications but endpoint-server scenarios have been poorly studied. In particular, no automatic solution to obtain a dynamic and efficient configuration for endpoints has been reported. They are still, then, mostly manually configured during the deployment phase. However, current Smart Homes present a very dynamic behaviour, where the information characteristics (entropy, generation rate etc.) can change dramatically in short periods.

The objective of this paper is, therefore, to describe an automatic and dynamic configuration algorithm for endpoints in Smart Homes, increasing (as much as possible) the communication efficiency at every moment. The paper proposes a definition for “communication efficiency” considering the most important costs associated to the operation of resource constrained endpoints and the value of the obtained information. This mathematical model is employed to do a prediction about the server behaviour and information characteristics, corrected with real measurements and Bayes theorem. Once it is detected the real server pattern, the endpoint modifies its configuration to increase the communication efficiency, according to the proposed model. Basically, two relevant configuration parameters are controlled: the endpoint lifecycle and the information compression method.

The structure of the paper is as follows: Section 2 presents the state of the art on configuration algorithms to improve efficiency in Smart Homes and resource constrained devices. Section 3 describes the main proposal, including the mathematical model and the configuration algorithm. Section 4 includes an experimental validation analysing the performance of the proposed solution. Finally, Section 5 shows the conclusions and future work.

## 2. State of the Art: Configuration Algorithms to Improve Efficiency in Smart Homes

Configuration process is a complex procedure in Smart Homes, so it is usually addressed at different independent levels. Typically, two types of works may be found in the context of Smart Homes [11]: papers about service configuration and discussions about network configuration.

Proposals for an automatic network configuration are the most common. Basically, all these works are focused on basic interoperability, so the final objective of those algorithms is the component installation in the Smart Home in an automatic manner [12]. To perform this installation operation some works employ configuration robots based on artificial intelligence [12], software defined network (SDN) technologies [13], new architectures with specific configuration middleware [14] or Domain Name Systems (DNS) [15]. Other works only discuss about security issues and other future challenges in relation to Smart Homes configuration [16]. None of these works, nevertheless, consider the system efficiency in the configuration process.

Network configuration solutions to improve the system efficiency are always focused on energy consumption. Some proposals are focused on efficient configuration solutions employing the minimum energy amount [17]. However, in these works, the focus is not the system operation but the configuration algorithm itself; thus, after an energy efficient configuration the system may operate very inefficiently. Only some very sparse proposals address the challenge of efficient operation, although limited to the use of the minimum required energy amount [18] and focused on sensor-server communications (through, for example, flexible configuration middleware controlling the network architecture). These works are usually known as “energy-efficient self-adaptation” solutions and sometimes are also applied to other similar technologies such as Internet of Things [19].

From another point of view, some authors address the Smart Homes automatic configuration at service level. Typical proposals at this level are based on virtual sensor representations [20] which are mapped into real deployments. Other works employ models and description languages to enable an automatic and dynamic system configuration [21]. Although, in general, these works do not consider the future efficient system operation, some authors refer to this requirement as a desired objective [21,22].

In that way, as a general conclusion, automatic and dynamic configuration solutions for Smart Homes or similar technologies (such as Smart Environments) are not focused on a future efficient system operation [23]. To address this challenge some specific system architectures [24] have been reported (using, for example, smart gateways) but they are focused (once more) on sensor networks and sensor-server communications.

In this work we propose, on the contrary, a complementary configuration algorithm which may operate together with any existing technology or architecture. Once endpoints are configured at network and service level (and then connectivity and interoperability are guaranteed), an algorithm to configure and probably modify, those characteristics affecting the system efficiency is periodically executed to improve, as much as possible, the resource usage.

## 3. Efficient Communications with Resource Constrained Information Endpoints in Smart Homes

In this section we define our understanding of “efficiency,” including all the costs we are considering affect this parameter. Then, probabilistic models for every cost are proposed and, finally, based on studies about the efficiency behaviour, we are describing our dynamic configuration solution.

### 3.1. Global Scenario and Efficiency Definition

In general, information endpoints, to be interoperable with different systems and technological solutions, include several configuration options. These options, as said, are usually manually selected during the endpoint deployment. However, current Smart Homes tend to present a very dynamic behaviour with real-time characteristics and permanent configurations do not enable efficient long-term communications. Changes in the type of content being sent to the endpoints or in the information renewal rate, may turn an initially very efficient configuration, into a total waste of resources. The solution, then, is to allow a dynamic and smart configuration mechanism to be continuously running in the endpoints.

We are assuming endpoints have been already configured at network and service level, guarantying the connectivity and interoperability. Moreover, server may (or not) support a negotiation process to adapt the endpoints and server’s behaviour.

**Figure 1:**
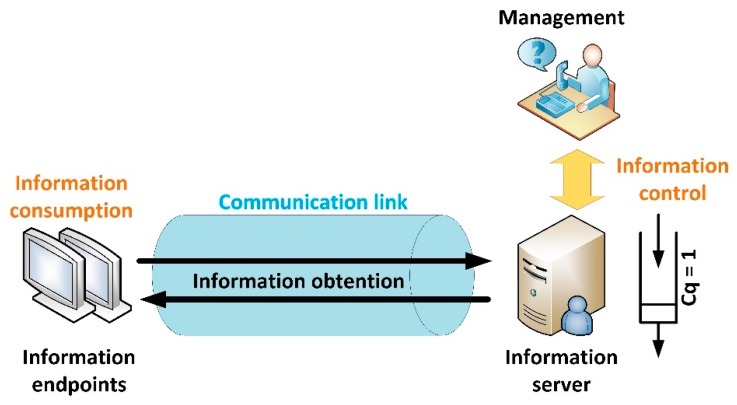
Architecture for a communication endpoint-server link in Smart Homes.

Figure 1 shows the basic architecture for a communication link between a server and an endpoint in a Smart Home.

As can be seen, content is managed by users, who can manually trigger an information actualization; or create automatic mechanisms through prosumer environments [25] to modify the system behaviour and information characteristics according to a collection of rules or situations. These information control orders are the final cause of inefficiencies in fixed configurations but they are external to any information system or solution, so they are not considered in this work. Thus, in our model, the information server embeds both, its own functionalities and behaviours inherited from managers.

In this context, the efficiency in a communication link between an endpoint and an information server $\eta_{com}$ is defined as the relation between the cost (value) of the consumed information $Q_{info}$ and the total invested cost to recover and consume that information $Q_{total}$ (1).

(1) $$\eta_{com}=\frac{Q_{info}}{Q_{total}}$$

Formally, efficiency is defined following an economic understanding: as the relation between the generated products (information blocks, in this case) and the resources invested to get those products (energy, memory and computing time in this case). For example, the relation between the memory required to compute an information block and the information it provides to users. To perform a fair comparison, both amounts are represented by their value or cost (both concepts are employed as synonyms). The cost (or value) of any resource or product is a dimensionless variable representing how essential or sparse the resource is, in the context of the communication link. For example, in endpoints with a low battery charge, the cost of the required current to refresh the displayed information is very high (as charge is sparse and remaining amount is totally essential for the endpoint’s operation).

As information blocks cannot be generated in a null time, for this analysis we are considering time as a discrete variable n, obtained from sampling the system situation every $T_{s}$ seconds. The value of $T_{s}$ is selected in such a way at least one information block can be generated during this period. The total (discrete) time employed to evaluate the system efficiency is $N_{study}$. If a longer operation time must be considered, it may be studied as a sequence of intervals.

Now, we are formally analysing all cost functions and values affecting the proposed efficiency rate, so global amounts are broken down in all elemental components. As different cost values represent the usage or different resources, to make comparable amounts, all costs are ranging in the interval [0,1] and are dimensionless. Thus, all cost calculation expressions include normalization parameters $\alpha_{i}$ to ensure those properties.

The cost (value) of the consumed information $Q_{info}$ is, in general, a function $C_{I}\left[\cdot \right]$ depending on the block generation time $n_{gen}$ and the time when the information is actually consumed (if so), $n_{con}$ (2). In general, as time passes, the value of an information block decreases. Although, as time passes, produced information blocks may contain more valuable information (for example, first samples from sensors are useless until a statistical equilibrium is reached and that also depends on the situation and on the sensor’s accuracy). All costs for all consumed blocks should be aggregated. Function $C_{I}\left[\cdot \right]$ is named as “cost function.”

(2) $$Q_{info}=\frac{1}{\alpha_{info}}\left(\underset{\begin{array}{c} \forall \;consumed\;block\\ b_{i} \end{array}}{\sum }C_{I}\left[n_{gen}^{b_{i}},\;n_{con}^{b_{i}}\right]\right)$$

This amount, nevertheless, may be also expressed as the balance between the (aggregated) original value of all generated information blocks, $Q_{info_{total}}$ and all lost values by those blocks, including non-consumed (lost in the server’s queue, see Figure 1) information blocks, $Q_{info_{lost}}$ and the degradation suffered by consumed blocks because of time evolution, $Q_{info_{deg}}$ (3).

(3) $$Q_{info}=\frac{1}{\alpha_{info}}\left(Q_{info_{total}}-Q_{info_{lost}}-Q_{info_{deg}}\right)$$

Cost of consumed information, although expressed as a balance between generated and lost cost (3), may be also calculated through cost functions (4). In this case, we are using a new cost function $C_{B}\left[\cdot \right]$, whose mathematical expression and meaning will be described in Section 3.2.

(4) $$Q_{info}=\frac{1}{\alpha_{info}}\left(\underset{\begin{array}{c} \forall \;produced\;block\\ b_{i} \end{array}}{\sum }C_{B}\left[b_{i}\right]-\underset{\begin{array}{c} \forall \;lost\;block\\ b_{i} \end{array}}{\sum }C_{B}\left[b_{i}\right]-\underset{\begin{array}{c} \forall \;consumed\;block\\ b_{i} \end{array}}{\sum }\left(C_{B}\left[b_{i}\right]-C_{I}\left[n_{gen}^{b_{i}},\;n_{con}^{b_{i}}\right]\right)\right)$$

With respect to the total invested cost $Q_{total}$, considered in the efficiency definition (1), three basic components are identified in our model: the link management cost $Q_{link}$, the information obtention cost $Q_{obten}$ and the information consumption cost, $Q_{consump}$ (5).

(5) $$Q_{total}=\frac{1}{\alpha_{total}}\left(Q_{link}+Q_{obten}+Q_{consump}\right)$$

The link management cost includes all effects and resource usage caused by the endpoint’s lifecycle (6). It includes costs associated to wake up process, $Q_{wake-up}$, sleep process, $Q_{sleep}$ and stand-by process, $Q_{stand-by}$. Costs associated to link establishment and shutdown are not considered, as these processes are run during the system deployment (or disassembly) and, then, cannot be considered as an operation cost.

(6) $$Q_{link}=\;\frac{1}{\alpha_{link}}\left(Q_{wake-up}+\;Q_{stand-by}+Q_{sleep}\right)$$

The cost of information obtention, $Q_{obten}$, includes basically two processes: the query procedure to check for new information in the server, with a cost $Q_{check}$ and the information recovery procedure, with a cost $Q_{recov}$. On the other hand, the information recovery cost is the aggregation of two different costs (7): the reception (or transmission) cost, $Q_{recep}$ and the decompression cost (if existing), $Q_{decom}$. Other costly processes such as encryption or authentication could be also considered but in this initial work we are focusing on a basic communication link configuration. Results and models might change if additional processes and variables are considered.

(7) $$Q_{obten}=\;\frac{1}{\alpha_{obten}}\left(Q_{check}+Q_{recov}\right)=\;\frac{1}{\alpha_{obten}}\left(Q_{check}+Q_{recep}+Q_{decom}\right)$$

The last cost to be analysed is the information consumption cost, $Q_{consump}$. This cost is directly related to the endpoints’ functionalities and procedures they must perform to consume the received information (if any) or to process that no new information is available (for example, to display the received image or refresh the existing one). This cost will increment each time an endpoint looks for new information, according to function Σ[·]. The cost to be added would be $Q_{update}$ or $Q_{no-update}$ depending on whether a new information block to be consumed is received or not (8).

(8) $$Q_{consump}=\;\Sigma \left[Q_{update},\;Q_{no-update}\right]$$

### 3.2. Information Model

In this work, information blocks (generated by the information server) are characterized by two main variables:

- Information block’s lifetime: As said, current Smart Homes are real-time solutions and then information has a very short lifetime. In particular, in real-time applications (such as video streaming) an information block has value until a new and more recent block is produced. Then, the old block gets valueless. Here, we are considering the same model. As a consequence, information server (see Figure 1) may be seen as a queue with unitary capacity ($C_{q}=1$). Thus, any information block is stored waiting for being transmitted, until it is sent or a new block is produced, when it is removed from the queue and replaced by the most modern block. Important exceptions to this model may be also found, as some information blocks may provide more durable information than others (for example, in video surveillance applications). However, in this work, we are not considering these “backward analysis applications” (which may be supported by storing all information blocks in a repository) but real-time solutions where information has value for a short period of time. Contrary to scenarios where sensors send data to servers, where information has a long lifetime but loses value (cost) as time passes [26] due to physical processes continue evolving; in the proposed scenario (Smart Homes), where endpoints receive information from servers, information keep the same value until it is totally replaced (valueless) by new information. This is an intuitive notion for images to be shown in displays, music, video, advertisements and so forth. In conclusion, for this analysis we are considering $Q_{info_{deg}}=0$, as consumed information blocks are not degraded as time passes.
- Information Shannon’s entropy, H: For an information block B codified with k-bit symbols, the Shannon’s entropy determines how many bits (in average) of these k bits per symbol provide information. Considering its mathematical expression (9), this entropy parameter is maximum (H=k bits) for totally random information blocks (equiprobable symbols); and minimum (H=0) for blocks where only one symbol is employed.
  (9) $$H\;\left(B\right)=-\;\underset{s_{i}\;\in \;B}{\sum }p\left(s_{i}\right)\log_{2}p\left(s_{i}\right)\;\;\;\;\;\;\;being\;\;s_{i}\;\;\;k-bit\;symbol$$

Using this measure (entropy) it is possible to define an objective cost function, to obtain the value (cost) of an information block $b_{i}$; that is, we can define function $C_{B}\left[\cdot \right]$. First, considering the block entropy and L as the block length (in k-bit symbols), we can calculate the amount of information, I, in any block (10). Then, as any cost must range between zero and the unit but the information amount in a block varies between zero and infinity, the cost function must be an exponential law to agree with both ranges (11). In this cost function we introduce a free parameter, τ, representing how fast or slow the block cost grows with the information amount in the block.

(10) $$I\left(b_{i}\right)=H\left(b_{i}\right)\cdot L$$

(11) $$C_{B}\left[b_{i}\right]=1-e^{-\frac{I\left(b_{i}\right)}{\tau }}=\;1-e^{-\frac{H\left(b_{i}\right)\cdot L}{\tau }}$$

In order to obtain the final information cost $Q_{info}$, we must estimate the number of generated and lost blocks. To perform this calculation, we must model before the endpoints and server behaviour. 

### 3.3. Information Endpoint and Server Models

Basically, four different endpoints are considered in this work, describing the most common and employed behaviours for devices in Smart Homes nowadays: (i) always-on model, (ii) fixed-period wake-up model, (iii) dynamic wake-up scheduling and (iv) exponential evolution wake-up.

Always-on model is a trivial scenario, where endpoints are always enabled and available to receive new information from the server. Thus, endpoints are always powered, connectable and ready to react to any request. As endpoints in this model never sleep, it is easy to see that for these endpoints $Q_{wake-up}=Q_{sleep}=0$. Besides, because endpoints never sleep, they do not have to query the server for new information after waking up. If any information is available, they just receive it. Then, $Q_{check}=0$.

**Figure 2:**
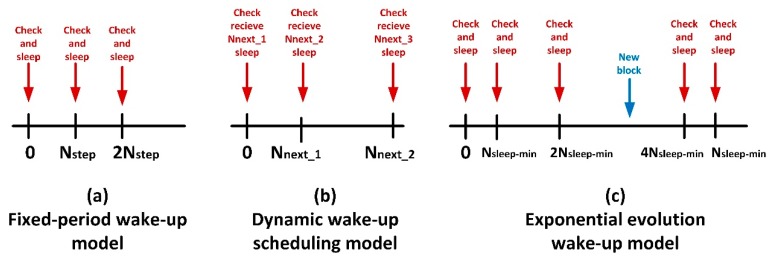
Lifecyle of endpoints according different behaviour models.

Fixed-period wake-up model is also an elemental scenario, although it considers the wake up and sleep procedures. Basically, each $N_{step}$ time instants the endpoint wakes up, connects to the information server looking for new information blocks, performs the corresponding actions and sleeps another time. Figure 2a shows a schematic chronogram describing this behaviour. Being $N_{sleep}$ the time the endpoint is sleeping, this parameter has a constant value in this case (12).

(12) $$N_{sleep}=N_{step}$$

The third model is more complicated. In the dynamic wake-up scheduling model, the information server is aware of the generation instant of the next information block, so it sends this datum together with the current information block to the endpoint. Then, the endpoint will sleep and wake up according to the received temporal scheduling. This model describes a wake-up scheduling that adapts the endpoint’s sleep time to the block generation rate. In this way, the endpoint automatically adapts to different patterns if the system behaviour changes. This wake-up scheduling, besides, aims to reduce inappropriate wake-up processes when there is not any new information pending in the server. See Figure 2b.

Finally, the exponential evolution wake-up model describes a behaviour which adapts the sleep period $N_{sleep}$ between checks for new blocks, exponentially increasing that time after each unsuccessful attempt to obtain new information from server, starting from a minimum value $N_{sleep-min}$ (13), see Figure 2c. The sleep time remains constant after reaching a certain maximum value $N_{sleep-max}\;$. Besides, after a successful attempt to obtain new information from the server, the sleep time is fixed another time to its minimum value $N_{sleep-min}$.

(13) $$N_{sleep}=\{\begin{array}{c} N_{sleep-min}\;\;\;\;\;\;if\;new\;block\;is\;recieved\;or\;\;n=0\\ 2\cdot N_{sleep}\;\;\;\;if\;no\;new\;block\;is\;recieved\;and\;N_{sleep}<\;N_{sleep-max}\\ N_{sleep-max}\;\;\;\;\;\;if\;no\;new\;block\;is\;recieved\;and\;N_{sleep}\geq \;N_{sleep-max} \end{array}$$

On the other hand, information server (which in our model embeds both managers’ functionalities and its own) presents a behaviour (in relation to information block creation) which may be modelled by a discrete stochastic process, S[s,n]. This process may take several different forms, representing each one a different information block creation pattern. However, in this paper, we are considering the three most common and relevant patterns: (i) predefined fixed pattern, (ii) stationary Bernoulli pattern and (iii) Poisson pattern.

In servers following a predefined fixed block creation pattern, the random variable s is a Bernoulli variable where only two different states are considered (14). One value (s=1) represents a situation where an information block is generated in the corresponding time instant. The other value represents the opposite case. In this model, only one information block (as maximum) may be generated per time instant. As the block creation pattern is predefined, the probability of each state is known a priori for each time instant and only takes values in the ${\mathbb{Z}}^{2}=\left\{0,1\right\}$ set. In particular, it is known the set $N_{new-block}$ (15) storing all time instants (14) for which $p_{pre}=\;p\left(s=1\right)=1.$ At any other instant $p_{pre}=0\;$(16).

(14) $$s=\left[\begin{array}{c} 1\\ 0 \end{array}\right]=\;\left[\begin{array}{c} new\;block\;in\;sever\\ no\;new\;block\;in\;server \end{array}\right]$$

(15) $$N_{new-block}=\left[n_{1}^{new},\;n_{2}^{new},\ldots ,n_{M}^{new}\right]$$

(16) $$S\left[s,n\right]=\{\begin{array}{c} p_{pre}=1\;\;\;\;\;\;\;if\;n\in \;N_{new-block}\\ p_{pre}=0\;\;\;\;\;\;\;if\;n\notin \;N_{new-block} \end{array}$$

On the other hand, servers presenting a stationary Bernoulli pattern have a similar behaviour and model but more general. In particular, the success probability $p_{ber}=\;p\left(s=1\right)$ is a real number in the interval [0,1], the same value for all time instants (17).

(17) $$S\left[s,n\right]=\{\begin{array}{c} p_{ber}=cte\;\;\;\;\;\;\;\forall \;n\;\;\;\;p_{ber}\;\epsilon \;\left[0,1\right]\\ q_{ber}=1-p_{ber}=cte\;\;\;\;\;\;\forall \;n \end{array}$$

Finally, servers following Poisson patterns are completely different. These servers are characterized by the generation of a certain mean number of information blocks $\lambda_{poission}$ each $N_{study}$ time units. In this case, besides, random variable s takes values in ℕ, the set of the natural numbers (18). It represents the number of blocks generated in a certain time instant.

(18) s∈ℕ≡{0,1,2,…}

Poisson servers are characterized by consecutive block generations distributed in time according to an exponential law. Statistic theory establishes that this behaviour corresponds to a Poisson distribution with mean value $\frac{\lambda_{poisson}}{N_{study}}\cdot n$ for each time instant (19).

(19) $$S\left[s,n\right]=Poi\left(\frac{\lambda_{poisson}}{N_{study}}\cdot n\right)=\frac{1}{s!}e^{-\frac{\lambda_{poisson}}{N_{study}}\;\cdot \;n}\cdot {\left(\frac{\lambda_{poisson}}{N_{study}}\cdot n\right)}^{s}$$

### 3.4. Information Cost Calculation

As said in Section 3.2, before obtaining the information cost, we must estimate the number of generated and lost blocks. These values, however, are random variables whose final expressions depend on the server’s and endpoint’s behaviours.

First, we are evaluating the number of generated information blocks, $M_{B}$. In this case, as generated blocks are only dependent on the server behaviour, the endpoint model does not have to be considered. Table 1 shows the obtained results for each server model.

Servers behaving according to a predefined fixed pattern generate a fixed amount of information blocks, equal to the number of time instants when an information block is predefined to be created. Nevertheless, the other two server models generate a random number of blocks, so the corresponding probability distribution is proposed. For Bernoulli servers, the probability distribution may be calculated as a set of $M_{B}$ independent successful events, between $N_{study}$ trials. Then, a binomial distribution describes this distribution. For Poisson servers, the number of generated blocks is described by a Poisson distribution.

Now, on the other hand, we must calculate the number of lost blocks, $M_{L}$. Table 2 shows the corresponding values or probability distributions (depending on the case) for all endpoint’s and server’s types. First, always-on endpoints receive information block just at the moment they are generated, so blocks are never lost. However, fixed-period wake-up endpoints (as well as the other two possible endpoint’s models) may be asleep while several blocks are generated and lost. Then, all blocks above the unit generated while endpoints are slept are lost (as only the newest information block has value, see Section 3.2). In general, therefore, $M_{L}$ information blocks will be lost if $M_{L}+1$ blocks are generated in $N_{sleep}$ seconds. For predefined fixed pattern servers, this amount is easily calculated considering the block creation pattern. Moreover, a special case is the “synchronization situation” for fixed pattern servers. In this configuration, it is selected the sleep period $N_{step}$ to guarantee there is not any lost block (20).

(20) $$N_{step}=min\left\{n_{k-1}^{new}-n_{k}^{new}\right\}\;\;\;k\in \mathbb{N},\;\;\;n_{k}^{new},\;n_{k-1}^{new}\in N_{new-block}\;$$

For other server models, being $M_{L}^{i}$ the number of lost blocks during the i-th sleep period of a device, it is easy to calculate the probability of each value of $M_{L}^{i}$ using the binominal or Poisson distribution (depending on the server type).

Besides, being $C_{sleep}$ the number of sleep periods in $N_{study}$ time units (21) and using a sequential decomposition process to analyse all possible lost block distributions in $C_{sleep}$ periods (and the corresponding probability) we can obtain the results shown in Table 2 for fixed-period wake-up endpoints.

(21) $$C_{sleep}=\frac{N_{study}}{N_{step}}$$

Dynamic wake-up scheduling endpoints are more difficult to study, as their behaviour is not predefined by any function. As said, in general, these endpoints adapt to the server behaviour. Then, we are assuming (hereinafter) their behaviour is as follows (previous works have proved these are the most efficient behaviours for each case [9]):

- For predefined fixed pattern servers dynamic wake-up scheduling endpoints are configured to follow the same server’s pattern (so there are not any lost blocks)
- For stationary Bernoulli servers, where all time instants have the same probability to generate an information block, it is selected (for this work) as the most profitable endpoint model a fixed-period wake-up model.
- Finally, for Poisson servers which generate information blocks according to an exponential law, it is selected (for this work) as the most profitable endpoint model an exponential evolution wake-up model.

Thus, considering previous assumptions, the number of lost blocks in each case may be obtained as explained for the corresponding endpoint model. 

Finally, we must analyse exponential evolution wake-up endpoints. In predefined fixed pattern servers, it is possible to calculate the exact number of lost blocks as no random component is affecting the result. For Bernoulli servers, as well as for Poisson servers, the probability distribution may be also calculated using a sequential decomposition process where $C_{exp}$ different time (sleep) periods are considered. As time periods have not a homogeneous duration, $C_{exp}$ is variable depending on each situation. Nevertheless, for clarity in the mathematical analysis (which in this case is pretty complex) we are considering in this initial work $C_{exp}$ is constant and its value is fixed to the mean value among the possible variance interval (22).

(22) $$C_{exp}\in \left[1,\ldots ,\frac{N_{study}}{N_{min}}\right]\;\Rightarrow \;E\left[C_{exp}\right]=\frac{N_{study}}{2\cdot N_{min}}=\;2^{r_{exp}}\cdot N_{min}$$

Now, the loss probability depends on the real duration of each sleep period, which depends on the duration and number of generated blocks in the previous period. Then, conditional probabilities appear (to be aggregated and obtain the final absolute value). Besides, the sleep period gets increased only if no block is generated during the previous period, whose probability is controlled by the server natural probability distribution $p_{n}$, following a Binomial (or Poisson) distribution. Besides, all probabilities are parametric on the sleep period length, including the absolute loss probability $p_{l}$, employed to obtain the conditional probabilities. Finally, we must consider the probability of a unique information block to be generated at each time (sleep) period, $p_{sucess}$, which is calculated aggregating all possible cases according to probability laws (23).

(23) $$p\left(A\right)=\underset{\forall B}{\sum }p(A\;|B)\cdot p\left(B\right)$$

The final expression deduction is a complex induction process and mathematical expressions turn especially large if recursive loops are removed but obtained results allow estimating in a very precise manner the lost block rate.

### 3.5. Link Management Cost Calculation

The link management cost is totally caused by device lifecycle. Basically, costs under this name (cost of wake-up $Q_{wake-up}$, stand-by $Q_{stand-by}$ and sleep $Q_{sleep}$ processes) are associated to an energy consumption. All (mobile) endpoints have an independent and limited-capacity battery with an available electrical charge BAT, measured in ampere-hour. As, in this work, we are considering time as a discrete variable, it is necessary to obtain first the battery charge in ampere-discrete time units (24).

(24) $$BAT\left(An\right)=\frac{BAT\left(Ah\right)}{1h}\cdot \frac{1h}{T_{s}}$$

Now, the cost of each charge unit should be variable and depend on the resting charge: as the battery is running out charge, the value of the resting energy grows up. Several different cost functions $C_{E}\;\left[\cdot \right]$ could be selected but all of them should fulfil the requirements described in Section 3.1 (cost ranges between zero and the unit). However, many different works [27,28] have proved the value of any resource goes up exponentially as it is sparser. Thus, we are also employing an exponential law as cost function (25a). In this case it is a function with memory as previous consumptions, $BAT_{con}^{i}$, affect the resting energy cost. This function may be also written in a memory-less form if a new parameter $BAT_{level}$ is considered (25b). In both cases, parameter τ (a real value) determines how fast the energy cost grows.

(25a) $$C_{E}=\;1-e^{-\;\frac{\frac{BAT}{BAT-\sum_{i}BAT_{con}^{i}}-1}{\tau }}$$

(25b) $$C_{E}\left[BAT_{level}\right]=\;1-e^{-\;\frac{\frac{BAT}{BAT-BAT_{level}}-1}{\tau }}\;\;\;\;\;being\;BAT_{level}=\;\underset{i}{\sum }BAT_{con}^{i}$$

Previously presented cost function enables us to determine the cost of each charge unit but some processes (such as stand-by) are running for several time units or consume higher current amounts. Then, the cost of a certain amount of charge units $E_{i}$ may be obtained integrating along the cost function (26).

(26) $$C_{E}\left[E_{i};\;BAT_{level}\right]=\int_{\frac{BAT}{BAT-BAT_{level}}}^{\frac{BAT}{BAT-\left(BAT_{level}+E_{i}\right)}}\;\left(1-e^{-\;\frac{x-1}{\tau }}\right)dx=\;A+\frac{BAT}{BAT-\left(BAT_{level}+E_{i}\right)}+\;\tau \cdot e^{-\;\frac{\frac{BAT}{BAT-\left(BAT_{level}+E_{i}\right)}\;-\;1}{\tau }}\;A=-\frac{BAT}{BAT-BAT_{level}}-\;\tau \cdot e^{-\;\frac{\frac{BAT}{BAT-BAT_{level}}\;-\;1}{\tau }}$$

Considering these mathematical expressions, we can now obtain the values for the three costs studied in this subsection. With respect to the wake-up process, we are considering it occurs in a one-time unit, consuming $I_{wake-up}$ amperes. Then, the unitary cost of each wake-up in any endpoint, $Q_{wake-up}^{unitary}$ may be easily calculated using the cost function (27). The same assumptions are applied to the sleep process, which consumes $I_{sleep}$ amperes; thus, the unitary cost of each sleep process $Q_{sleep}^{unitary}$ is also easily calculated (28). $BAT_{level}$ parameter must be fixed dynamically depending on the endpoint’s situation.

(27) $$Q_{wake-up}^{unitary}\left(BAT_{level}\right)=C_{E}\left[I_{wake-up}\cdot 1;\;BAT_{level}\right]$$

(28) $$Q_{sleep}^{unitary}\left(BAT_{level}\right)=C_{E}\left[I_{sleep}\cdot 1;\;BAT_{level}\right]$$

While endpoints are slept, they do not consume energy, so no cost must be considered. However, while endpoints are on, a stand-by cost appears. While endpoints are in a stand-by state they consume $I_{stand-by}$ amperes. Considering the endpoint is on for a $N_{stand-by}$ length period, the unitary cost of a stand-by period $Q_{stand-by}^{unitary}$ may be calculated through the cost function (29). For endpoints which are on only to look for updates, no stand-by cost is produced.

(29) $$Q_{stand-by}^{unitary}\;\left(N;BAT_{level}\right)=C_{E}\left[I_{stand-by}\cdot N;\;BAT_{level}\right]$$

Then, for different endpoint’s models, the global costs associated to wake-up and sleep processes and stand-by periods are different. Table 3 shows the calculation expressions for each case. We are assuming at n=0 batteries are totally charged. 

For always-on endpoints, only the cost associated to the permanent stand-by state must be considered. For other endpoint’s models no stand-by cost must be calculated. However, wake-up and sleep processes generate other costs to be obtained. In particular, the number of sleep periods for each model was calculated in Section 3.4. Therefore, it is enough to consider an aggregation of unitary costs, considering for each sleep period that one sleep and one wake-up processes occur. Besides, the battery charge decreases linearly according to consumed current for each process, so it is simple to obtain the battery level at each moment.

### 3.6. Information Obtention Cost Calculation

Once evaluated the loss probability and the link management cost, we must address the cost associated to information obtention (considering the server has a new information block to be sent). This cost, $Q_{obten}$, is the composition of two partial costs: query process cost, $Q_{check}$ and the recovery process cost, $Q_{recov}$.

The query process cost refers the usage of hardware resources such as communications modules. Each query has a unitary cost, $Q_{check}^{unitary}$. As the elements affecting this cost are not consumable, we are assuming it has a constant value selected according to the endpoint implementation (we are not addressing hardware details in this work). Besides, as always-on endpoints never sleep, they never query the server for new blocks: if any block is available, the server just sends it. Table 4 shows the cost calculation for the different endpoint models, considering the number of sleep periods (a query process is performed after wake-up).

Now, the information recovery cost is not related to endpoints’ characteristics but to communication system’s configuration. This cost is the aggregation of two amounts: the information reception cost, $Q_{recep}$ and the decompression cost, $Q_{decom}$.

With respect to the information reception cost, we are assuming a unitary cost describing the cost of receiving a bit, $Q_{recep}^{unitary}$. This cost includes spectrum reservation, reception buffers and so forth. Then, for a received information block with a length of L h-bit symbols (see Section 3.2), the reception cost may be easily obtained (30).

(30) $$Q_{recep}=L\;\cdot h\;\cdot \;Q_{recep}^{\mathrm{unitary}}$$

Now, to analyse the decompression cost we must consider different compression methods. In this work we take into account three different algorithms: (i) Run-length encoding (RLE), (ii) Huffman-Qopt method (where Q is a parameter indicating the number of symbols to be employed in compressed blocks) and (iii) no compression (raw transmission). All decompression methods are modelled as the following sequence: a compressed symbol is read and then the equivalent decompressed symbol is written. This sequence is repeated until the entire message is read. Mathematically, then, the decompression cost may be modelled as an algebraic operation (31).

(31) $$Q_{decom}=\left(h\cdot Q_{read}^{unitary}+k\cdot Q_{write}^{unitary}\cdot L_{symbol}^{decompressed}\right)\cdot L_{compressed}+Q_{RAM}\;$$

In this expression, different unitary costs are considered referring the consumed processing time: the unitary cost of reading a bit $Q_{read}^{unitary}$ and the unitary cost of writing a bit, $Q_{write}^{unitary}$. In general, compressed symbols are h-bit symbols, where k≠h. Besides, $L_{compressed}$ refers to the compressed information block length in h-bit symbols; and $L_{symbol}^{decompressed}$ refers to the number of k-bit symbols obtained from a compressed h-bit symbol. Finally, $Q_{RAM}$ is the cost of all transitory data structures (in volatile memory) needed to perform the decompression process. Considering an endpoint has a RAM memory with capacity RAM, the cost may be calculated using a cost function $C_{M}\left[\cdot \right]$ (32) isomorphic to the one described for energy cost (26) and considering $RAM_{con}$ the consumed memory amount.

(32) $$C_{M}=1-e^{-\;\frac{\frac{RAM}{RAM-RAM_{con}}-1}{\tau }}$$

For raw transmission the decompression cost is zero, $Q_{decom}=0$, as no compression method is employed. Besides, RLE does not require any transitory data structure, so $Q_{RAM}=0$. Table 5 shows the relation between the compressed and raw length of an information block $b_{i}$, depending on its entropy. These expressions may be deducted considering how the different algorithms work [9]. For Huffman Q-optimum algorithm $H_{min}^{Q+1}\left(b_{i}\right)$ represents the minimum entropy for a block $b_{i}$, calculated among all possible dictionaries with Q+1 symbols.

In these expressions, randomness is embedded by block’s entropy, which is at the end a stochastic term. 

### 3.7. Information Consumption Cost Calculation

Finally, once an information block is received by an endpoint (or not), it must be consumed and operations related to actualization, decision and device management must be taken. These operations have a cost $Q_{consump}$. Basically, two different costs may appear each time an endpoint looks for new information blocks:

- If server has in the queue any new information block, the endpoint (after obtaining it) must consume it (for example, display the retrieved image). Later, memory must be cleaned, timers programmed and so forth. All this process has a cost $Q_{update}$.
- On the other hand, if no new block is available, the endpoint only must refresh the existing information, program timers and so forth. All these operations have a cost, $Q_{no-update}$, which is usually lower than the cost including the new information processing. In always-on endpoints, this cost is not applicable as they develop an alternative stand-by cycle.

For server following a deterministic behaviour, then, $Q_{consump}$ may be directly calculated. For servers with a random behavior (Poisson and Bernoulli servers), a probability distribution is obtained. Table 6 shows the obtained expression for each case.

First, we must consider that, contrary to other costs, information consumption cost $Q_{consump}$ cannot take any real or integer value. Only values decomposable as a combination of $Q_{update}$ and $Q_{no-update}$ costs are possible values (33). Additional limits could be applied for certain combinations of server and endpoint models.

(33) $$Q_{consump}=M_{1}\cdot Q_{update}+M_{2}\cdot Q_{no-update}\;\;\;\;being\;M_{1},M_{2}\;\in \;\mathbb{N}$$

Now, for always-on endpoints, it is easy to obtain the value (or probability distribution) of the global cost, as only an amount of $Q_{update}$ units must be added each time the server generates a new information block. Equally, for fixed-period wake-up endpoints, it is only needed to evaluate the probability of generating, at least, one information block in each sleep period. That may be done directly using the Binomial or Poisson distribution, depending on the server type (Bernoulli or Poisson).

As said in Section 3.5, dynamic wake-up scheduling endpoints follow other models depending on the server type, so no new calculation is required.

Finally, exponential evolution wake-up endpoints require larger and more complex discussions. When employed with predefined fixed pattern servers, a deterministic calculation may be done. However, when employed Poisson or Bernoulli servers it is necessary to consider the Bayes laws (23) to obtain the global probability distribution. This mathematical development has been also employed in other subsections, although in this case a binary probability $p_{b}$ is defined, to calculate the natural probability of a Bernoulli (and Poisson) distribution to generate any amount of information blocks or (in the opposite case) not generate any block.

The last problem we must address in this subsection is the calculation of partial costs $Q_{update}$ and $Q_{no-update}$. Basically, these costs refer to the usage of hardware components such as microprocessors, RAM memory, displays and so forth. A combination of different cost functions depending on the endpoint implementation should be employed. In this initial work, as we are not addressing implementation particularities, we are assuming these two partial costs have a predefined value representing the resource consumption according to the endpoint hardware.

### 3.8. Proposed Algorithm for Optimizing Communication Efficiency

In order to propose an optimization algorithm in a reasoned manner, we firstly analyse the behaviour of the communication efficiency according to some relevant parameters using the previously described models. A multi-dimensional analysis would be required to consider all possible cases and situations but this approach cannot be implemented in practice for more than two parameters. Therefore, we are focusing our analysis on those parameters the endpoints may change or negotiate: the endpoint’s lifecycle and the compression method.

**Figure 3:**
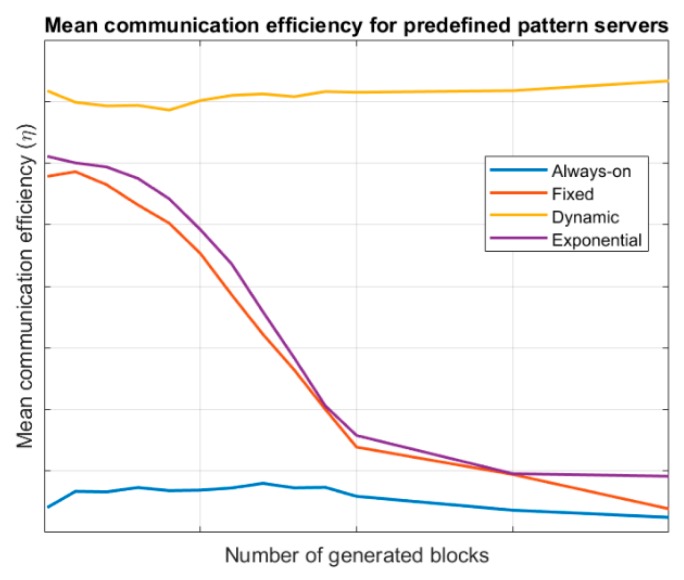
Mean communication efficiency for predefined pattern servers.

Figure 3 shows the evolution of the mean communication efficiency for the different endpoint’s lifecycles, considering a server following a predefined pattern. Results are evaluated for different values of $card\left\{N_{new-block}\right\}$ (number of generated information blocks). Specific values for communication efficiency depend on many variables at this point but our objective is only a comparative study between the different endpoint’s lifecycle; thus, no values are shown in axes in Figure 3. As can be seen, dynamic wake-up scheduling endpoints present the best efficiency, as their lifecycle is always synchronized with the servers, reducing the information losses and the link management and information consumption costs. Both, fixed-period wake-up model and exponential evolution wake-up endpoints present a variable behaviour as costs tend to be similar but information losses grow as more blocks are generated. Anyway, exponential evolution wake-up endpoints are slightly better as they can adapt, increasing or reducing the sleep period. Always-on endpoints present a low efficiency 

**Figure 4:**
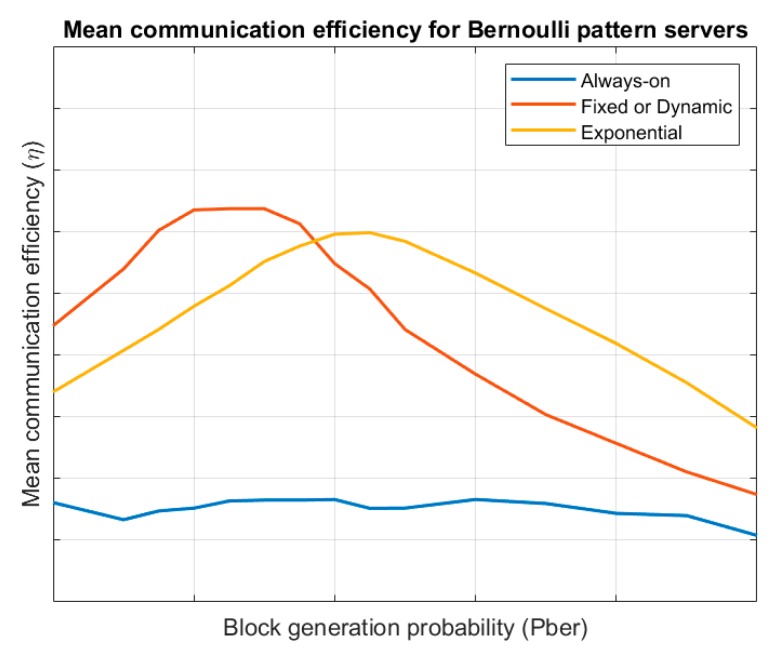
Mean communication efficiency for Bernoulli pattern servers.

Figure 4 shows the shows the evolution of the mean communication efficiency for the different endpoint’s lifecycles, considering a server following a Bernoulli pattern. Results are evaluated for different values of the block generation probability $p_{ber}$. Always-on endpoints present a similar efficiency to other cases but fixed-period wake-up model and exponential evolution wake-up endpoints have a variable behaviour. For low block generation probabilities, the link management cost is much bigger than the value of the obtained information and efficiency is low. On the other hand, for large block generation probabilities the information losses go up and efficiency is also low. For values in the middle, a balance is reached and efficiency is maximum. Besides, for low block generation probabilities, fixed-period wake-up endpoints are more efficient and for high block generation probabilities exponential evolution wake-up endpoints are better.

**Figure 5:**
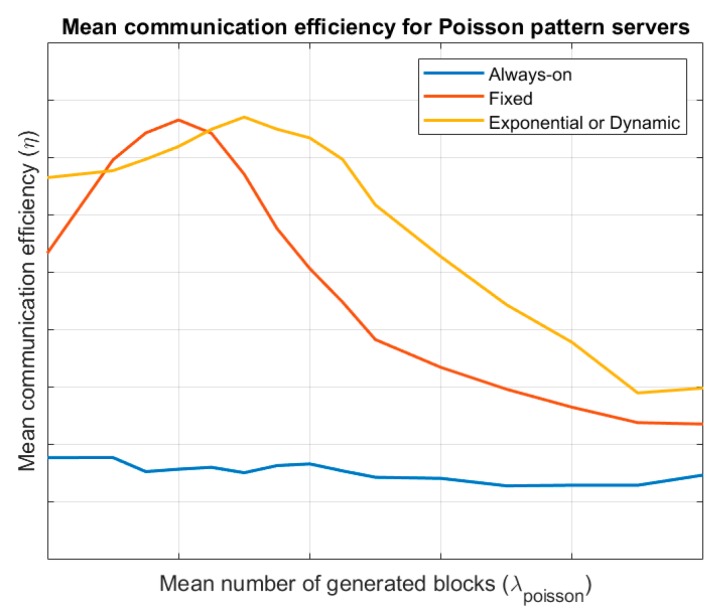
Mean communication efficiency for Poisson pattern servers.

Finally, Figure 5 shows the evolution of the mean communication efficiency for the different endpoint’s lifecycles, considering a server following a Poisson pattern. Results are evaluated for different values of the mean number of generated blocks $\lambda_{poisson}$. It is similar to that obtained for Bernoulli servers, although in this case (globally), exponential evolution wake-up endpoints are more efficient.

**Figure 6:**
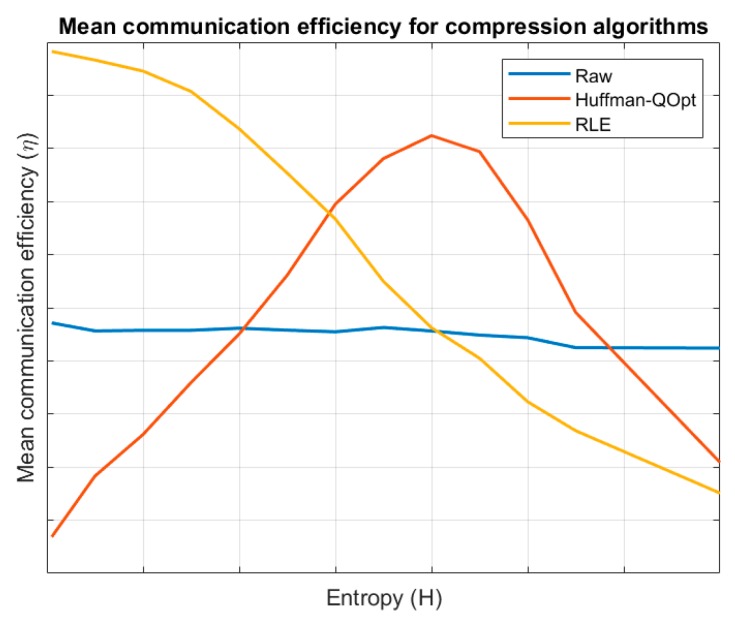
Mean communication efficiency for different compression algorithms.

On the other hand, we are evaluating the system efficiency depending on the compression algorithm. Figure 6 shows the obtained results for different values of entropy. For low entropy information blocks, RLE algorithm is very efficient but as entropy grows the efficiency goes down. The same behaviour is shown for Huffman algorithm but it is a very costly algorithm (code is very complex and large and large transitory data structures are needed) and only for a small number of situations its use is efficient. For high entropy information blocks the transmission in raw format is finally more efficient.

Then, considering all showed results, the proposed algorithm follows some rules according the previous figures. Namely:

- By default, all endpoints follow an always-on lifecycle and using a raw transmission system. This approach, although it is the least efficient, allow us to collect information about the system behaviour in the fastest manner, so a more efficient configuration may be easily selected.
- If low entropy information blocks are detected, the RLE compression algorithm will be selected. For high entropy blocks, raw transmission will be employed; and for intermedium situation the Huffman QOpt algorithm will be configured. Considering Figure 6 and the fact that entropy ranges in the interval [0, k], Table 7 shows the proposed operation limits.
- For predefined pattern servers, a dynamic wake-up scheduling will be negotiated with the server. If it is not available, an exponential evolution wake-up model will be employed.
- For Bernoulli pattern servers, two different situations are clearly shown (see Figure 4). For low values of the block creation probability (for example, $p_{ber}<0,5$), the fixed-period wake-up model is more efficient. In any other case, the exponential evolution wake-up model will be employed by endpoints.
- Finally, for Poisson pattern servers, an exponential evolution wake-up model will be employed by endpoints.

Now, in order to detect the server configurations and, then, change dynamically the endpoints’ configurations to increase as much as possible the communication efficiency, we are using the Bayes theorem (34). It is important to note that server may not be aware about certain behaviours inherited from managers (such as the information generation rate).

(34) $$p\left(server=PAT\;|\;Q_{i}\right)=\frac{p\left(\;Q_{i}\;|\;server=PAT\;\right)}{\sum_{\forall \;T}p\left(\;Q_{i}\;|\;server=PAT\;\right)\cdot p\left(server=PAT\;\right)}p\left(server=PAT\right)$$

All endpoints may easily calculate the described costs, $Q_{i}$ (such as the information value $Q_{info}$) considering the proposed cost functions and the real resource consumption and/or number of received information blocks. Then, after collecting data for a certain time period, it is possible (using previously described expressions) to evaluate the probability of the server to follow a certain pattern PAT, that is, $p\left(\;Q_{i}\;|\;server=PAT\right)$, known the costs $Q_{i}$. Thus, using the Bayes theorem and the proposed expression (34) it is evaluated the “posteriori” probability of a server to follow the pattern PAT. For this calculation we are considering all server’s pattern equally probable, so $p\left(server=PAT\right)=\frac{1}{3}$.

As three different costs related to the server pattern have been defined ($Q_{info}$, $Q_{link}$ and $Q_{consump}$), three different probabilities will be obtained. Each probability will be mapped into three different integer numbers, according to two thresholds $p_{min}$ and $p_{max}$ (35).

(35) $$G\left(p_{i}\right)=\{\begin{array}{l} 1\;\;\;\;\;\;if\;\;\;p_{i}<p_{min}\\ 2\;\;\;\;\;\;if\;\;\;p_{min}\leq p_{i}\leq p_{max}\\ 3\;\;\;\;\;\;if\;\;\;p_{i}>p_{max} \end{array}$$

Finally, an aggregated estimator will be employed (36) to rank all possible server patterns. The pattern with a higher mark is selected as the real pattern. Only patterns with a mark above the global threshold $G_{threshold}$ will be considered in the decision process.

(36) $$G_{total}=G\left(p\left(server=PAT\;|\;Q_{info}\right)\right)\cdot G\left(p\left(server=PAT\;|\;Q_{link}\right)\right)\cdot G\left(p\left(server=PAT\;|\;Q_{consump}\right)\right)$$

In order to select the most efficient compression algorithm it is enough to evaluate the entropy of the received information blocks. If the central server allows stablishing a negotiation process, the compression method will be changed according to previously indicated rules. On the contrary, no change will be applied.

In order to avoid transitory effects (temporary behaviour that are not stable nor permanent), the same result must be obtained in several different and independent evaluations, $N_{eval}$, across $N_{hist}$ sequential evaluations, to change the endpoint’s configuration. Once efficiency is above a certain threshold $\eta_{threshold}$ the dynamic configuration process stops and will be run again if the efficiency monitoring procedure detects efficiency goes down.

Algorithm 1 codifies the described behaviour.


**Algorithm 1: Dynamic Communication Efficiency Optimization**
**Input:** Set of received information blocks B
   Set of battery consumptions $BAT_{con}^{i}$
   Estimated costs $Q_{\mathrm{consump}}$ and $Q_{\mathrm{obten}}$ (hardware dependent)    Circular buffer BUFF with $N_{hist}$ last $G_{PAT-max}$ evaluations   Circular buffer ENT with $N_{hist}$ last $\bar{H\left(B\right)}$ evaluations**Output:** Endpoint configurationCalculate $Q_{info}$ for the set of received information blocks B, $Q_{info}=C_{I}\left[B\right]$
Calculate $Q_{link}$ using the set of battery consumptions $BAT_{con}^{i}$, $Q_{link}=C_{E}\left[BAT_{con}^{i}\right]$
Calculate the mean entropy of the information blocks, $\bar{H\left(B\right)}$ Obtain the mean communication efficiency $\bar{\eta }=\eta \left(Q_{info},Q_{link},\;Q_{\mathrm{obten}},Q_{\mathrm{consump}}\right)$
**if**$\bar{\eta }<\;\eta_{threshold}$ or endpoint is not configured **then**   $CONF_{selected}=ALWAYS-ON$   **for** every server pattern PAT∈{predefined, Bernoulli, Poisson}
**do**      **for** every cost $Q_{i}\in \;\left\{Q_{info},\;Q_{link},\;Q_{obten},\;Q_{consump}\right\}$
**do**         Calculate $p_{i}=\;p\left(\;Q_{i}\;|\;server=PAT\;\right)$ using the proposed mathematical model         Obtain $p_{PAT,\;i}=\;p\left(server=PAT\;\;|\;Q_{i}\right)$ using Bayes theorem      **end for**      Calculate $G_{PAT-total}\;=\underset{i}{\prod }G\left(p_{PAT,\;i}\right)\;$
   **end for**   Calculate $G_{PAT-max}=max\;\left\{G_{PAT-total},\;\;\;being\;G_{PAT-total}>\;G_{threshold}\right\}\;$
   Insert $G_{PAT-max}$ in BUFF
   **if**
$G_{predefined-max}$ is contained in BUFF at least $N_{eval}$ times **then**       **if** server allows dynamic scheduling **then**            $CONF_{selected}=DYNAMIC$       **else**            $CONF_{selected}=EXPONENTIAL$       **end if**   **else if**
$G_{Bernoulli-max}$ is contained in BUFF at least $N_{eval}$ times **then**       Estimate $p_{ber}$ using the set of received information blocks B
       **if**
$p_{ber}<0,5$ then           $CONF_{selected}=FIXED$       **end if**   **else**       **if**
$G_{Poisson-max}$ is contained in BUFF at least $N_{eval}$ times **then**          $CONF_{selected}=EXPONENTIAL$       **end if**   **end if**   Insert $\bar{H\left(B\right)}$ in ENT
   **if** server allows negotiation **then**       **if**
$\bar{H\left(B\right)}\;\in \left[0,\frac{k}{2}\right]$ in ENT at least $N_{eval}$ times **then**          COMPRESSION=RLE       **else if**
$\bar{H\left(B\right)}\;\in \left[\frac{k}{2},3\frac{k}{2}\right]$ in ENT at least $N_{eval}$ times **then**          COMPRESSION=HUFFMAN       **else**          **if**
$\bar{H\left(B\right)}\;\in \left[3\frac{k}{2},k\right]$ in ENT at least $N_{eval}$ times **then**             COMPRESSION=RAW          **end if**      **end if**   **end if**
**end if**


## 4. Experimental Validation and Results

In order to evaluate the performance and validate the proposed solution, in this section it is described an experimental validation based on simulation scenarios and a first real implementation.

### 4.1. Experiment Description

Four experiments were carried out. The first group includes the three initial experiments and are based on simulation scenarios and tools. The second group, including only the fourth and final experiment, employs as main element an initial real implementation of the proposed solution. 

Simulation scenarios and experiments are built using the NS3 network simulator. NS3 is a research simulation tool where scenarios and networks are described using C++ language. Results are obtained as a discrete sequence of events which may be processed and analysed after finishing the simulation. This network simulator considers three basic elements: information sources, communication networks and information endpoints. As seen, these elements perfectly fit the elements in our scenario (see Figure 1).

All simulations are carried out using a Linux architecture (Linux 16.04 LTS) with the following hardware characteristics: Dell R540 Rack 2U, 96 GB RAM, two processors Intel Xeon Silver 4114 2.2G, 2TB SATA 7.2K rpm.

Basically, the first three experiments are performed in the same scenario. A Smart Home, where connectivity is supported by Wi-Fi solutions and where only one information source emulating the server behaviour and functions is considered. The number of endpoints in the same scenario is variable, as well as the characteristics of content generated by the information source. All elements are considered to be configured at network and service level, to guarantee the connectivity and interoperability. 

In order to implement the proposed configuration algorithm in the endpoints and obtain relevant results, endpoints are connected to virtual instances running over the same operating system. These virtual machines are created and maintained through LXC technologies (Linux Containers) and the libvirt interface which enable the automatic creation and monitoring of these instances. Containers execute a unique process consisting of the described solution in Algorithm 1. Using ghost nodes and TAP bridges the output and inputs of these virtual instances is connected to the simulation elements representing the endpoints. In that way, it is possible to evaluate the performance of the proposed solution and enrich our simulation with real information. All the virtual machines are monitored in the use of their resources through the libvirt interface in order to feed the proposed algorithm with these data.

Using this scheme three different experiments were carried out. The first one considers fifteen endpoints in the scenario. Seven endpoints are receiving content with a low entropy (text) and eight endpoints are receiving data with a high entropy (images). The experiment studies the efficiency evolution in the proposed scenarios for the two different endpoints groups. Three cases are considered. In each case the server behaviour is changed: predefined fixed pattern, Bernoulli pattern and Poisson pattern. For each case, twelve simulations were developed and presented results are the mean of all obtained realizations. One hundred operation hours are simulated in each case.

The second experiment analyses the delay required by our solution to react and change the endpoints’ configuration to the most efficient scheme after a spontaneous change in the server or information characteristics. To perform this analysis, fifteen endpoints receiving all of them the same information are considered. Three cases are considered: change in the server pattern, change in the information entropy, change in the server pattern and information entropy at the same time. For each case, twelve simulations were developed and presented results are the mean of all obtained realizations. One hundred operation hours are simulated in each case.

The third experiment, the last one using simulation tools, is focused on comparing the proposed solution to existing proposals in the state of the art. The efficiency reached by the proposed solution is compared to the efficiency reached by a standard solution [11]. The same scenario than in second experiment was employed. Five hundred simulations were performed for each algorithm and maximum reached efficiency was measured. 

**Figure 7:**
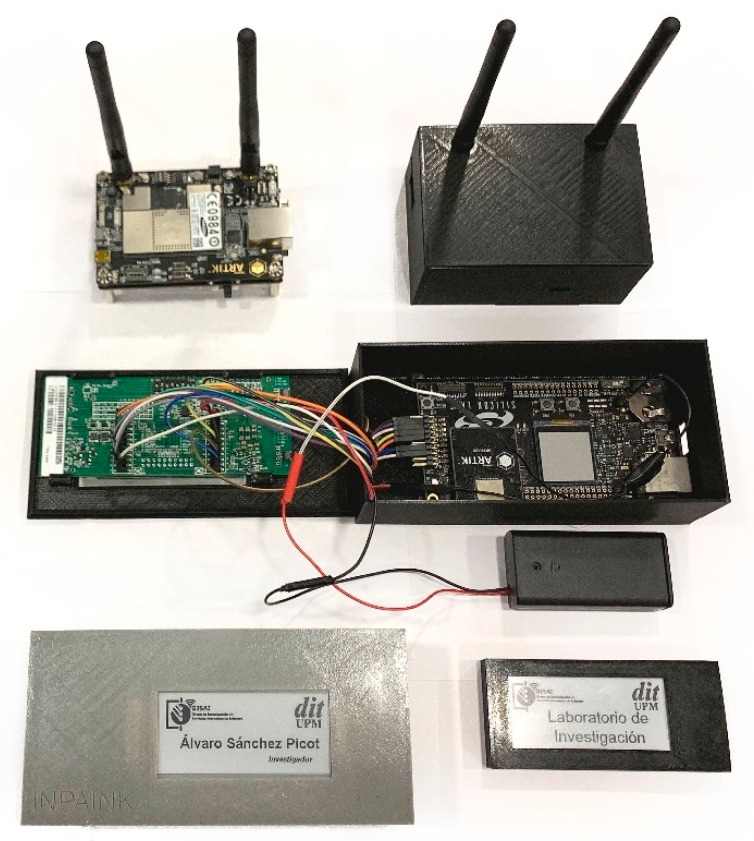
First real implementation of information endpoints for Smart Homes using the proposed configuration algorithm.

The four experiment is quite different. In order to evaluate the performance of the proposed solution in a real deployment, it is developed a first initial system implementation. The proposed Smart Home consisted of a central server where a web server generated the information blocks. Then, five information endpoints were connected to this server thought a Smart Gateway implemented using the Samsung Artik 530 (Linux) architecture. Endpoints are electronic ink displays, where images are shown. These endpoints are based on Artik 020 architecture and connected through Bluetooth wireless technology to the Smart Gateway. Figure 7 shows the described deployment.

Artik 020 architecture is based on a high Performance 32-bit 40 MHz ARM Cortex^®^-M4 with DSP instruction and floating-point unit for efficient signal processing. It also includes a 256 kB flash program memory and a 32 kB RAM data memory. Using these resource-constrained devices it is evaluated the real consumption caused by the proposed solution. A very important aspect in our proposal is the possibility of the algorithm to be implemented and executed in resource constrained endpoints. The described deployment was operated for three days and data about the resource consumption was collected through the debugging interface. In particular, the use of data memory (RAM), program memory (flash) and processing time is evaluated. The resource consumption was evaluated for different situations and configuration actions.

Finally, for all four experiments, the Table 8 represents the value of all the configuration parameters described in the mathematical model. 

### 4.2. Results

**Figure 8:**
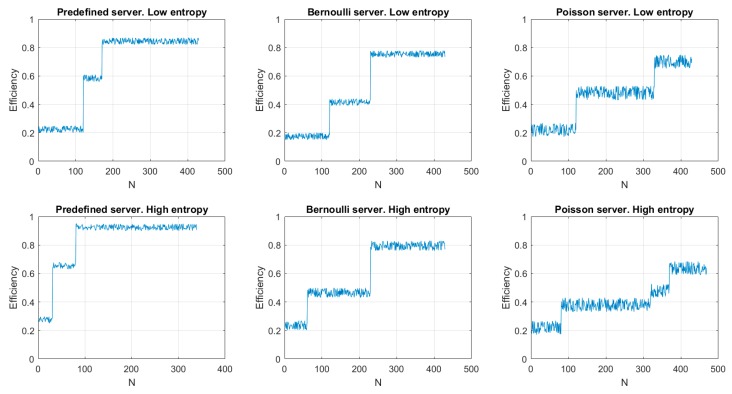
First experiment: results.

Figure 8 shows the results of the first experiment. As can be seen, the efficiency evolves according to a staircase function. This is caused by the double analysis described in our proposal: first the endpoint lifecycle and later the compression algorithm. In fact, calculations associated to compression algorithms (i.e., the information entropy calculation) are more stable in time, so the most efficient compression algorithm is selected much faster than the most efficient lifecycle, whose analysis includes many random variables.

Thus, the first step in the stair corresponds to the compression method selection and the second step to the lifecycle selection. Anyway, as can be seen, the proposed algorithm increases the efficiency operation above 60% in all cases. Even, for predefined servers (where the analysis considers few variables and then statistical noise is less relevant) efficiency reaches up to 90% (approximately). On the other hand, as Poisson servers are studied with expressions where probabilistic variables have a higher weight, statistical noise in this case is higher and present greater fluctuations. Besides, in this case, time required to obtain the most appropriate lifecycle is also higher than in any other case (and minimum for predefined servers where probabilistic variables have a smaller impact). On the other hand, in endpoints receiving messages with a high entropy, the time required to select the most efficient compression method is much smaller than in endpoint receiving low entropy messages. That is caused by the behaviour of entropy function (logarithmic) which is more stable as the independent variable goes up. Finally, in some situations (see “Poisson server, high entropy” figure) temporary states may appear caused by false convergences (which are lately corrected).

**Figure 9:**
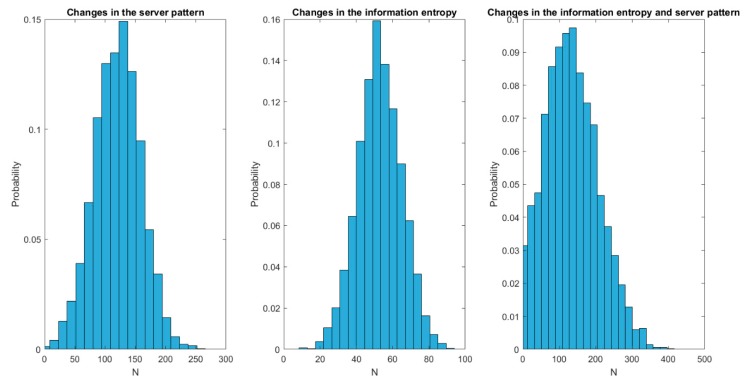
Second experiment: results.

Figure 9 shows the results of the second experiment. As can be seen, changes in the information blocks’ entropy are solved much faster than any other change; approximately 50% faster than changes in the server pattern. In standard time, changes in the information entropy are addressed in, approximately, 50 (fifty) seconds, while changes in the server pattern require around one hundred twenty (120) seconds. Dispersion (jitter) is also higher for changes in the server pattern, although it is especially relevant for situations when server patterns and information entropy suffer changes at the same time. In particular, situations where both changes occur together present a dispersion 100% higher than any other situation.

**Figure 10:**
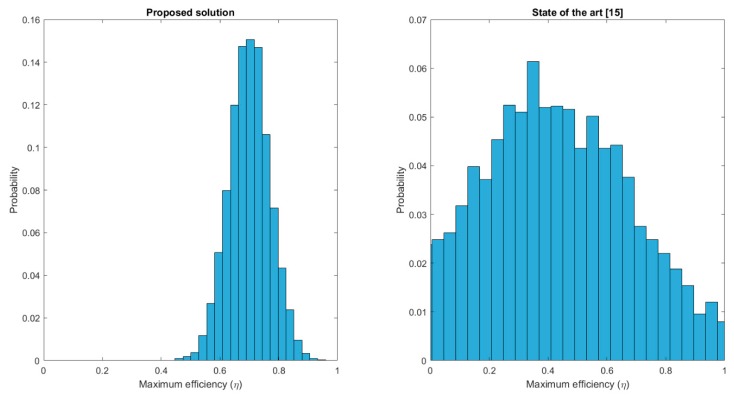
Third experiment: results.

Figure 10 presents the results of the third experiment. Maximum reached efficiency is evaluated for different simulations, showing an increase up to 70% when employing the proposed solution. However, the main difference is the probability distribution for each case. Solutions in the state of the art are not focused on a future efficient operation, so the maximum reached efficiency is a totally random value with a uniform distribution and almost every possible value has a non-null probability. On the contrary, using the proposed solution, maximum efficiency is a gaussian distribution with a quite low dispersion and centred around $\eta_{max}=0.7$ (approximately). As can be seen, it is a very relevant improvement.

Table 9 shows the results of the fourth and last experiment. As can be seen, the size of the program, although relevant, it is acceptable for resource constrained endpoints. Moreover, the use of memory RAM is low (below 20%), which fits the requirements of endpoints in Smart Homes. Besides, the consumed processing time is always below three (3) seconds per actualization (execution).

## 5. Conclusions and Future Work

In this work we propose a new configuration algorithm for endpoints in Smart Homes, so that they can operate in the most efficient way according to the dynamic characteristics of received information blocks and central server behaviour. The proposed algorithm makes predictions using a mathematical model, where all involved costs in the information reception and consumption are identified and quantified. Different server patterns and information block types (presenting different entropies) are considered in the model, to select the most appropriate endpoint lifecycle and compression method to increase efficiency as much as possible.

Predictions are corrected using the Bayes theorem with real measurements about the real resource consumption. The proposed solution is complementary to any other installed configuration solution to guarantee the system connectivity and interoperability.

In order to validate the proposed solution an experimental validation was carried out using simulation scenarios and real deployments. Results show a good performance of the proposed solution and a relevant efficiency increase in the system operation in comparison to previous proposals.

As future work, more complex models should be considered and a unique general expression for all possible server patterns should be created. Besides, more advanced artificial intelligence instruments could be integrated into the described algorithm to replace the Bayes theorem which may be limited for certain situations (for example, when hidden patterns govern the system behaviour).

## Figures and Tables

**Table 1:** Total number of generated blocks in the proposed scenario. Random variables.

Server Model	Probability Distribution
Predefined fixed pattern	$M_{B}=card\left\{N_{new-block}\right\}$
Stationary Bernoulli pattern	$p\left(M_{B}\right)=\left(\begin{array}{c} N_{study}\\ M_{B} \end{array}\right){\left(p_{ber}\right)}^{M_{B}}{\left(q_{ber}\right)}^{N_{study}-M_{B}}\;\;M_{B}\leq \;N_{study}$
Poisson pattern	$p\left(M_{B}\right)=\;\frac{1}{M_{B}!}e^{-\lambda_{poisson}}\cdot {\left(\lambda_{poisson}\right)}^{M_{B}}$

**Table 2:** Total number of lost blocks in the proposed scenario. Random variables.

Server Model	Endpoint Model	
Predefined fixed pattern	Always-on	$M_{L}=0$
	Fixed-period wake-up model	$M_{L}=\underset{\begin{array}{c} \forall \;r\;\in \;N\;\;\\ r\cdot N_{step}\;\leq \;N_{study} \end{array}}{\sum }\left(card\left\{\left[n_{k}^{new},\;k\in \mathbb{N},\;\left(r-1\right)\cdot N_{step}\leq n_{k}^{new}<r\cdot N_{step}\right]\right\}-1\right)$ $M_{L}^{syn}=0$
	Dynamic wake-up scheduling	$M_{L}=0$
	Exponential evolution wake-up	$M_{L}=\;\underset{i}{\sum }M_{L}^{i}$ $M_{L}^{i}\;max.\;integer\;number\;such\;that\;\;n_{k_{i}-1}^{new}-n_{k_{i}}^{new}>N_{min}\;and$ $n_{k_{i}-1}^{new}-n_{k_{i}+M_{L}^{i}}^{new}>2^{r}\cdot N_{min}\;\;and\;n_{k_{i}-1}^{new}-n_{k_{i}+M_{L}^{i}}^{new}<2^{r+1}\cdot N_{min}$
Stationary Bernoulli pattern	Always-on	$M_{L}=0$
	Fixed-period wake-up model	$p\left(M_{L}\right)=\overset{M_{L}}{\underset{r_{0}=0}{\sum }}\overset{r_{0}}{\underset{r_{1}=0}{\sum }}\cdots \overset{r_{C_{sleep}-1}}{\underset{r_{C_{sleep}}=0}{\sum }}p\left(M_{L}^{0}=M_{L}-r_{0}\right)\cdot \left(\overset{r_{C_{sleep}}}{\underset{i=1}{\prod }}p\left(M_{L}^{i}=r_{i-1}-r_{i}\right)\right)\;\cdot p\left(M_{L}^{C_{sleep}}=r_{C_{sleep}}\right)$ $p\left(M_{L}^{i}=M\right)=\left(\begin{array}{c} N_{step}\\ M+1 \end{array}\right){\left(p_{ber}\right)}^{M+1}{\left(q_{ber}\right)}^{N_{step}-\;M-1}\;\;\;\;\;1\leq M\leq \;N_{step}-1$
	Dynamic wake-up scheduling	
	Exponential evolution wake-up	$p\left(M_{L}\right)=\overset{M_{L}}{\underset{r_{0}=0}{\sum }}\overset{r_{0}}{\underset{r_{1}=0}{\sum }}\cdots \overset{r_{C_{exp}-1}}{\underset{r_{C_{exp}}=0}{\sum }}p_{l}\left(M_{L}^{0}=M_{L}-r_{0}\;;\;N_{min}\right)\cdot \left(\overset{r_{C_{exp}}}{\underset{i=1}{\prod }}p\left(M_{L}^{i}=r_{i-1}-r_{i}\;\;|\;M_{L}^{i-1}\right)\right)\;\;\;\;\;\;\;\;\cdot p\left(M_{L}^{C_{exp}}=r_{C_{exp}}\;|\;M_{L}^{C_{exp}-1}\right)$ $\begin{array}{l} p\left(M_{L}^{k}=\;M\;|\;M_{L}^{k-1}\right)\\ =\{\begin{array}{c} \overset{k}{\underset{i\;=0}{\sum }}p_{l}\left(M_{L}^{k}=\;M\;;\;\;2^{i}\cdot N_{min}\right)(\overset{i-1}{\underset{r=0}{\prod }}p_{n}\left(0\;;\;2^{r}\cdot N_{min}\right))p_{success}\left(k-1-i\right)\;\;\;if\;\;\;\;M_{L}^{k-1}=0\\ p_{l}\left(M_{L}^{k}=\;M\;;\;\;N_{min}\right)\;\;\;\;\;\;if\;\;\;\;M_{L}^{k-1}\neq 0 \end{array} \end{array}$ $p_{l}\left(M_{L}^{i}=\;M\;;\;N\right)=\left(\begin{array}{c} N\\ M+1 \end{array}\right){\left(p_{ber}\right)}^{M+1}{\left(q_{ber}\right)}^{N-\;M-1}\;\;\;\;\;1\leq M\leq \;N-1$ $p_{n}\left(M\;;\;N\right)=\left(\begin{array}{c} N\\ M \end{array}\right){\left(p_{ber}\right)}^{M}{\left(q_{ber}\right)}^{N-\;M}\;\;\;\;\;0\leq M\leq \;N$ $\begin{array}{c} p_{success}\left(k\right)=\overset{k}{\underset{r_{0}=0}{\sum }}\overset{k-r_{0}-1}{\underset{r_{1}=0}{\sum }}\overset{k-2-\left(r_{0}+r_{1}\right)}{\underset{r_{2}=\;0}{\sum }}\cdots \overset{1-\left(r_{0}+\cdots +r_{k-2}\right)}{\underset{r_{k-1}=0}{\sum }}\overset{k-1}{\underset{i=0}{\prod }}[p_{n}(1\;;\;\;2^{r_{i}}\\ \;\;\;\cdot N_{min})(\overset{r_{i}-1}{\underset{z=0}{\prod }}p_{n}\left(0\;;\;2^{z}\cdot N_{min}\right))]p_{n}\left(1\;;\;N_{min}\right) \end{array}$
Poisson pattern	Always-on	$M_{L}=0$
	Fixed-period wake-up model	$p\left(M_{L}\right)=\overset{M_{L}}{\underset{r_{0}=0}{\sum }}\overset{r_{0}}{\underset{r_{1}=0}{\sum }}\cdots \overset{r_{C_{sleep}-1}}{\underset{r_{C_{sleep}}=0}{\sum }}p\left(M_{L}^{0}=M_{L}-r_{0}\right)\cdot \left(\overset{r_{C_{sleep}}}{\underset{i=1\;}{\prod }}p\left(M_{L}^{i}=r_{i-1}-r_{i}\right)\right)\cdot p\left(M_{L}^{C_{sleep}}=r_{C_{sleep}}\right)$ $p\left(M_{L}^{i}=M\right)=\frac{1}{\left(M+1\right)!}e^{-\lambda_{poisson}\cdot \frac{N_{step}}{N_{study}}}\cdot {\left(\lambda_{poisson}\cdot \frac{N_{step}}{N_{study}}\right)}^{M+1}\;\;\;M\geq 1$
	Dynamic wake-up scheduling	$p\left(M_{L}\right)=\overset{M_{L}}{\underset{r_{0}=0}{\sum }}\overset{r_{0}}{\underset{r_{1}=0}{\sum }}\cdots \overset{r_{C_{exp}-1}}{\underset{r_{C_{exp}}=0}{\sum }}p_{n}\left(M_{L}^{0}=M_{L}-r_{0}\;;\;N_{min}\right)\cdot \left(\overset{r_{C_{exp}}}{\underset{i=1\;}{\prod }}p\left(M_{L}^{i}=r_{i-1}-r_{i}\;\;|\;M_{L}^{i-1}\right)\right)\;\;\;\;\;\;\;\;\;\cdot p\left(M_{L}^{C_{exp}}=r_{C_{exp}}\;|\;M_{L}^{C_{exp}-1}\right)$ $p\left(M_{L}^{k}=M\;|\;M_{L}^{k-1}\right)\;=\{\begin{array}{c} \overset{k}{\underset{i\;=0}{\sum }}p_{l}\left(M_{L}^{k}=\;M\;;\;\;2^{i}\cdot N_{min}\right)(\overset{i-1}{\underset{r=0}{\prod }}p_{n}\left(0\;;\;2^{r}\cdot N_{min}\right))p_{success}\left(k-1-i\right)\;\;\;if\;\;\;\;M_{L}^{k-1}=0\\ p_{l}\left(M_{L}^{k}=\;M\;;\;\;N_{min}\right)\;\;\;\;\;\;if\;\;\;\;M_{L}^{k-1}\neq 0 \end{array}$ $p_{l}\left(M_{L}^{i}=\;M\;;\;\;N\right)=\frac{1}{\left(M+1\right)!}e^{-\lambda_{poisson}\cdot \frac{N}{N_{study}}}\cdot {\left(\lambda_{poisson}\cdot \frac{N}{N_{study}}\right)}^{M+1}\;\;\;M\geq 1$ $p_{n}\left(M\;;\;N\right)=\frac{1}{M!}e^{-\lambda_{poisson}\cdot \frac{N}{N_{study}}}\cdot {\left(\lambda_{poisson}\cdot \frac{N}{N_{study}}\right)}^{M}\;\;\;M\geq 0$ $\begin{array}{r} p_{success}\left(k\right)=\;\overset{k}{\underset{r_{0}=0}{\sum }}\overset{k-r_{0}-1}{\underset{r_{1}=0}{\sum }}\overset{k-2-\left(r_{0}+r_{1}\right)}{\underset{r_{2}=\;0}{\sum }}\cdots \overset{1-\left(r_{0}+\cdots +r_{k-2}\right)}{\underset{r_{k-1}=0}{\sum }}\overset{k-1}{\underset{i=0}{\prod }}[p_{n}(1\;;\;\;2^{r_{i}}\\ \cdot N_{min})(\overset{r_{i}-1}{\underset{z=0}{\prod }}p_{n}\left(0\;;\;2^{z}\cdot N_{min}\right))]p_{n}\left(1\;;\;N_{min}\right) \end{array}$
	Exponential evolution wake-up	

**Table 3:** Link management costs for different endpoint’s models.

Endpoint Model		Cost		
		$Q_{wake-up}$	$Q_{stand-by}$	$Q_{sleep}$
**Always-on**		0	$Q_{stand-by}^{unitary}\;\left(N_{study};0\right)$	0
**Fixed-Period Wake-up Model**		$\overset{C_{sleep}}{\underset{i=1}{\sum }}Q_{wake-up}^{unitary}\left(\left(i\;-1\right)I_{wake-up}\right)$	0	$\overset{C_{sleep}}{\underset{i=1}{\sum }}Q_{sleep}^{unitary}\left(\left(i\;-1\right)I_{sleep}\right)$
**Dynamic Wake-up Scheduling**	**Bernoulli Server**		0	
	**Poisson Server**	$\overset{C_{exp}}{\underset{i=1}{\sum }}Q_{wake-up}^{unitary}\left(\left(i\;-1\right)I_{wake-up}\right)$		$\overset{C_{exp}}{\underset{i=1}{\sum }}Q_{sleep}^{unitary}\left(\left(i\;-1\right)I_{sleep}\right)$
**Exponential Evolution Wake-up**			0	

**Table 4:** Query process cost for different endpoint’s models.

Endpoint model		Cost $Q_{check}$
**Always-on**		0
**Fixed-Period Wake-up Model**		$C_{sleep}\;\cdot \;Q_{check}^{\mathrm{unitary}}$
**Dynamic Wake-up Scheduling**	**Bernoulli Server**	
	**Poisson Server**	$C_{exp}\;\cdot \;Q_{check}^{\mathrm{unitary}}$
**Exponential Evolution Wake-up**		

**Table 5:** Query process cost for different endpoint’s models.

Compression Method	Message Length (Raw) k-bit Symbols	Message Length (Compressed) h-bit Symbol $L_{compressed}$	Number of k-bit Symbols Per Compressed Symbol $L_{symbol}^{decompressed}$
Raw	L	L (h=k)	1
RLE	L	$\frac{L}{\left(2^{h-k}-1\right)-\left(2^{h-k}-2\right)\cdot \frac{H\left(b_{i}\right)}{k}\;}$	$\left(2^{h-k}-1\right)-\left(2^{h-k}-2\right)\cdot \frac{H\left(b_{i}\right)}{k}$
Huffman-Qopt	L	$\frac{L}{h}\cdot \frac{k}{k_{Q}}\cdot H_{min}^{Q+1}\left(b_{i}\right)$ $k_{Q}=\;\log_{2}\left(Q+1\right)$ $\begin{array}{l} H_{min}^{Q+1}\left(b_{i}\right)\\ =\underset{Dictionary\;Q+1}{min}\left(H\left(b_{i}\right)\right) \end{array}$	$\left(\frac{h-k_{Q}}{k}\right)H\left(b_{i}\right)+k_{Q}$

**Table 6:** Information consumption cost in the proposed scenario. Random variables.

Server Model	Endpoint Model	
Predefined fixed pattern	Always-on	$Q_{consump}=\;card\left\{N_{new-block}\right\}\cdot Q_{update}$
	Fixed-period wake-up model	$Q_{consump}=\;\overset{C_{sleep}}{\underset{i=1}{\sum }}Q_{step}\left(i\right)$ $Q_{step}\left(i\right)=\;\{\begin{array}{c} Q_{update}\;\;\;\;\;\;if\;\;\;\;\exists \;n_{k}^{new}\in N_{new-block}\;\;\vdots \;\;\;\left(i-1\right)N_{step}\;\leq n_{k}^{new}<i\cdot N_{step}\\ Q_{no-update}\;\;\;\;\;\;\;\;\;\;else \end{array}$
	Dynamic wake-up scheduling	$Q_{consump}=\;card\left\{N_{new-block}\right\}\cdot Q_{update}$
	Exponential evolution wake-up	$Q_{consump}=\;\underset{N_{new-block}}{\sum }Q_{update}+\;M\cdot Q_{no-update}$ $M\;integer\;number\;such\;that\;\;n_{k_{i}-1}^{new}-n_{k_{i}}^{new}>N_{min}\;and\;$ $n_{k_{i}-1}^{new}-n_{k_{i}+r}^{new}>2^{M-1}\cdot N_{min}\;\;and\;n_{k_{i}-1}^{new}-n_{k_{i}+r}^{new}<2^{M}\cdot N_{min}$
Stationary Bernoulli pattern	Always-on	$p\left(Q_{consump}=M\cdot Q_{update}\right)=\;\left(\begin{array}{c} N_{study}\\ M \end{array}\right){\left(p_{ber}\right)}^{M}{\left(q_{ber}\right)}^{N_{study}-\;M}$
	Fixed-period wake-up model	$p\left(Q_{consump}=M_{1}\cdot Q_{update}+M_{2}\cdot Q_{no-update}\right)\;\;\;\;\;\;\;=\left(\begin{array}{c} C_{sleep}\\ M_{1} \end{array}\right){\left(p_{success}\right)}^{M_{1}}{\left(1-p_{success}\right)}^{C_{sleep}-M_{1}}$ $p_{success}=\overset{N_{step}}{\underset{k=1}{\sum }}\left(\begin{array}{c} N_{step}\\ k \end{array}\right){\left(p_{ber}\right)}^{k}{\left(q_{ber}\right)}^{N_{step}-\;k}\;being\;M_{1},M_{2}\;\in \;\mathbb{N}\;\;\;\;M_{1}+M_{2}=C_{sleep}$
	Dynamic wake-up scheduling	
	Exponential evolution wake-up	$p\left(Q_{consump}=M_{1}\cdot Q_{update}+M_{2}\cdot Q_{no-update}\right)\;\;\;\;\;\;\;=\left(\begin{array}{c} C_{exp}\\ M_{1} \end{array}\right){\left(p_{success}\right)}^{M_{1}}{\left(1-p_{success}\right)}^{C_{exp}-M_{1}}$ $p_{success}=\overset{k_{exp}\;}{\underset{r_{0}=0}{\sum }}\overset{k_{exp}-1-r_{0}}{\underset{r_{1}=0}{\sum }}\overset{k_{exp}-2-\left(r_{0}+r_{1}\right)}{\underset{r_{2}=\;0}{\sum }}\cdots \overset{1-\left(r_{0}+\cdots +r_{k_{exp}-2}\right)}{\underset{r_{k_{exp}-1}=0}{\sum }}\overset{k_{exp}-1}{\underset{i=0}{\prod }}\left[p_{b}\left(\;2^{r_{i}}\;\;\;\;\;\;\cdot N_{min}\right)\left(\overset{r_{i}-1}{\underset{z=0}{\prod }}1-p_{b}\left(2^{z}\cdot N_{min}\right)\right)\right]p_{b}\left(N_{min}\right)$ $being\;M_{1},M_{2}\;\in \;\mathbb{N}\;\;\;\;M_{1}+M_{2}=C_{exp}$ $p_{b}\left(N\right)=\overset{\infty }{\underset{k=1}{\sum }}\left(\begin{array}{c} N\\ k \end{array}\right){\left(p_{ber}\right)}^{k}{\left(q_{ber}\right)}^{N-\;k}$
Poisson pattern	Always-on	$p\left(Q_{consump}=M\cdot Q_{update}\right)=\left(\begin{array}{c} N_{study}\\ M \end{array}\right){\left(p_{success}\right)}^{M}{\left(1-p_{success}\right)}^{N_{study}-M}$ $p_{success}=\overset{\infty }{\underset{k=1}{\sum }}\frac{1}{k!}e^{-\frac{\lambda_{poisson}}{N_{study}}}\cdot {\left(\frac{\lambda_{poisson}}{N_{study}}\right)}^{k}$
	Fixed-period wake-up model	$p\left(Q_{consump}=M_{1}\cdot Q_{update}+M_{2}\cdot Q_{no-update}\right)\;\;\;\;\;\;\;\;=\left(\begin{array}{c} C_{sleep}\\ M_{1} \end{array}\right){\left(p_{success}\right)}^{M_{1}}{\left(1-p_{success}\right)}^{C_{sleep}-M_{1}}$ $p_{success}=\overset{\infty }{\underset{k=1}{\sum }}\frac{1}{k!}e^{-\frac{\lambda_{poisson}}{N_{study}}N_{step}}\cdot {\left(\frac{\lambda_{poisson}}{N_{study}}N_{step}\right)}^{k}$ $being\;M_{1},M_{2}\;\in \;\mathbb{N}\;\;\;\;M_{1}+M_{2}=C_{sleep}\;$
	Dynamic wake-up scheduling	$p\left(Q_{consump}=M_{1}\cdot Q_{update}+M_{2}\cdot Q_{no-update}\right)\;\;\;\;\;\;\;=\left(\begin{array}{c} C_{exp}\\ M_{1} \end{array}\right){\left(p_{success}\right)}^{M_{1}}{\left(1-p_{success}\right)}^{C_{exp}-M_{1}}\;$ $p_{success}=\;\overset{k_{exp}\;}{\underset{r_{0}=0}{\sum }}\overset{k_{exp}-1-r_{0}}{\underset{r_{1}=0}{\sum }}\overset{k_{exp}-2-\left(r_{0}+r_{1}\right)}{\underset{r_{2}=\;0}{\sum }}\cdots \overset{1-\left(r_{0}+\cdots +r_{k_{exp}-2}\right)}{\underset{r_{k_{exp}-1}=0}{\sum }}\overset{k_{exp}-1}{\underset{i=0}{\prod }}\left[p_{b}\left(\;2^{r_{i}}\;\;\;\;\;\;\;\cdot N_{min}\right)\left(\overset{r_{i}-1}{\underset{z=0}{\prod }}1-p_{b}\left(2^{z}\cdot N_{min}\right)\right)\right]p_{b}\left(N_{min}\right)\;$ $being\;M_{1},M_{2}\;\in \;\mathbb{N}\;\;\;\;M_{1}+M_{2}=C_{exp}$ $p_{b}\left(N\right)=\overset{\infty }{\underset{k=1}{\sum }}\frac{1}{k!}e^{-\;\frac{\lambda_{poisson}}{N_{study}}\;\cdot \;N}\cdot {\left(\frac{\lambda_{poisson}}{N_{study}}\cdot N\right)}^{k}\;\;\;\;\;$
	Exponential evolution wake-up	

**Table 7:** Application limits for each compression algorithm.

Compression Algorithm	Application Limits
RLE	$H\left(b_{i}\right)\;\in \left[0,\frac{k}{2}\right]$
Raw	$H\left(b_{i}\right)\;\in \left[3\frac{k}{2},k\right]$
Huffman QOpt	$H\left(b_{i}\right)\;\in \left[\frac{k}{2},3\frac{k}{2}\right]$

**Table 8:** Configuration parameters for the experimental validation.

Parameter	Value
$N_{step}$	10
$\lambda_{poission}$	50
$N_{study}$	5000
$p_{ber}$	0.7
$T_{s}$	1 s
$N_{min}$	2
k	8
h	1
BAT	7200 mAh

**Table 9:** Fourth experiment: results.

Configuration Action	Use of RAM	Use of Program Space	Processing Time to Perform an Actualization
Predefined server to Bernoulli server	16%	34%	2.2 s
Predefined server to Poisson server	18%	34%	1.9 s
Bernoulli server to Poisson server	18%	34%	1.9 s
Entropy increasing	12%	34%	1.5 s
Entropy decreasing	12%	34%	1.5 s
